# Latent Class Analysis Offers Insight into the Complex Food Environments of Native American Communities: Findings from the Randomly Selected OPREVENT2 Trial Baseline Sample

**DOI:** 10.3390/ijerph17041237

**Published:** 2020-02-14

**Authors:** Brittany Wenniserí:iostha Jock, Karen Bandeen Roche, Stephanie V. Caldas, Leslie Redmond, Sheila Fleischhacker, Joel Gittelsohn

**Affiliations:** 1 School of Human Nutrition, Centre for Indigenous Peoples’ Nutrition and Environment (CINE), McGill University, Ste-Anne-de-Bellevue, QC H9X 3V9, Canada; 2 Johns Hopkins Bloomberg School of Public Health, Baltimore, MD 21205, USA; kbandee1@jhu.edu (K.B.R.); jgittel1@jhu.edu (J.G.); 3 University of North Texas, Denton, TX 76203, USA; svcaldas@gmail.com; 4 University of Alaska Anchorage, Anchorage, AK 99508, USA; lcredmond@alaska.edu; 5 Georgetown University Law Center, Washington, DC 20001, USA; sheilafly9@gmail.com

**Keywords:** food environment, Native Americans, obesity

## Abstract

Native Americans (NAs) experience a high burden of obesity and diabetes, yet previous research has not holistically described the unique food environments of NA communities. The objective of this paper is to describe the subgroups and demographic characteristics related to NA household food environments. Surveys collected food getting, food assistance, and sociodemographic variables from randomly selected adults from three NA communities (*n* = 300) in the Midwest and Southwest. Exploratory latent class analysis (LCA) identified the appropriate number of subgroups based on indicator responses. After assigning participants to classes, demographic differences were examined using bivariate analyses. NA household food environments could be described using two subgroups (“lower” and “higher access household food environments”). The “lower access” group had significantly higher age, smaller household size, and fewer children per household than the “higher access” group, while body mass index (BMI) did not significantly vary. This is the first LCA of NA household food environments and highlights the need for approaches that characterize the complexity of these environments. Findings demonstrate that NA household food environments can be described by developing subgroups based on patterns of market and traditional food getting, and food assistance utilization. Understanding NA household food environments could identify tailored individual and community-level approaches to promoting healthy eating for NA Nations.

## 1. Introduction

The majority of Native American adults (81%) were not at a healthy weight in 2008 [1] compared to 34% of the United States (US) general population [2], and 25% of Native American adults live at the poverty level compared to 13% of the total US population [3]. Native Americans have the highest prevalence of diabetes of any racial/ethnic group in the US [4]. These increased obesity and diabetes rates among Native Americans have occurred alongside a “nutrition transition” from traditional foods; typically from diets that were high in fiber and lean meats and daily life that was highly active, to one that is increasingly dependent on processed foods high in sugar, fat, and meat products and highly sedentary [5,6]. Reducing health disparities and advancing health equity for Native Americans requires intensive efforts to address the social determinants of health. The Healthy People 2020 Approach to Social Determinants of Health summarizes five areas for health promotion: economic stability, education, social and community context, health and health care, and neighborhood and built environment [7]. 

Previous research has shown mixed results in describing the association between the local food environment and obesity, with most studies finding no association [8]. However, few studies have rigorously explored the local food environment and obesity among Native American Nations, who endured significant land loss as a result of the Indian Removal Act of 1830 [9]. Existing research suggests that the majority of Native American reservations can be characterized as having low access to healthy foods [10]. That is, 77% of tribal households lived more than one mile from a supermarket, compared to 41% of all US households [10]. A mixed methods study examining Native American food environments in California found that there were significantly less healthy food outlets on or near reservations and that tribal members experienced many barriers in getting healthy food [11]. Previous research has demonstrated that convenience stores are the most common food outlet on reservations and that healthy foods are less available [12,13] and more expensive on-reservation compared to off-reservation [12]. Greater reliance on convenience stores and gas stations as primary food sources limits healthy food and beverage options [14] and has been associated with a greater prevalence of both obesity and diabetes [15]. 

Another important but understudied facet of Native American household food environments is the United States Department of Agriculture (USDA) Food Distribution Program for Indian Reservations (FDPIR), which provides food assistance to 276 rural Native American Nations and to an average of 90,083 Native Americans each month who may not have access to the USDA Supplemental Nutrition Assistance Program (SNAP) and to the USDA Special Supplemental Nutrition Program for Women, Infants, and Children (WIC) authorized retailers [16]. 

Nonetheless, reliance on food store access or federal food and nutrition assistance participation data alone provides an incomplete assessment of Native American food environments on-reservation, given that traditional food systems are used and valued part of Nations’ cultural identities [14]. A literature review of food environment measures by McKinnon et al. highlighted the need for statistical methods to describe the complex food environments of low-income, rural, and minority populations [17]. This study aims to address these knowledge gaps by describing patterns of perceived household food environments of diverse Native American communities. We hypothesized that individuals within the participating Native American communities would be comprised of multiple homogeneous and distinct subgroups, characterized by different patterns of access to and use of household food environment indicators. We aimed to further describe these subgroups by comparing the distribution of sociodemographic variables and hypothesized that the subpopulations underlying these subgroups would have different body mass index (BMI) distributions.

## 2. Materials and Methods

Study participants were part of the baseline assessment of the Obesity Prevention Research and Evaluation of interVention Effectiveness in NaTive North Americans 2 (OPREVENT2) study, a cluster-randomized controlled trial evaluating the impact of multi-level, multi-component obesity prevention programs among six Native American communities in the Midwest and Southwest US (clinical trial registration: NCT02803853; 10 June 2016) [18]. Participants of the OPREVENT2 baseline surveys were recruited from a list of randomly-selected tribal members from each community until at least 100 participants from each community (*n* = 600) were recruited. Tribal members of this list were eligible to participate in surveys if they provided signed consent; were between 18 and 75 years of age; considered themselves part of the community; lived in the community for ≥30 days; planned to live in the community for the next 18 months; were not pregnant or breastfeeding; did not participate in OPREVENT2 community member workshops; and self-identified as either the main food preparer or shopper for their household. Surveys were collected from September 2016 to May 2017. All participating communities provided tribal approval for the study. For the purposes of this study, a subset of the total OPREVENT2 baseline dataset was used, based on data use agreements with three of the communities, leaving a sample of 300 participants from three OPREVENT2 communities from the Midwest and Southwest regions. The research, instruments, and consent forms were approved by the Johns Hopkins School of Public Health Institutional Review Board (7027, Approved 25 May 2016), the Indian Health Service IRB (N16-N-04, Approved 20 May 2016), and the Navajo Nation Human Research Review Board (NNR-16-245, Approved 17 May 2016). 

Baseline OPREVENT2 surveys gathered information regarding sociodemographic variables and food getting and assistance. Sociodemographic variables included participant age, sex, household size, number of children per household, employment status, highest attained education level, marital status, and anthropometry. Anthropometry data included measured height (inches) and weight (pounds). Height and weight were measured at least twice, and a third measurement was taken if the height measurements differed by more than 0.5 inch and if the weight measurements differed by more than five pounds. Average height and weight measurements were used to calculate BMI.

We conceptualized the household food environment as being indicated by food assistance participation and healthy food getting of both traditional and market food items. Six food assistance questions (Appendix B) assessed household participation in food assistance programs (e.g., WIC, SNAP, and FDPIR), and respondents answered with a dichotomous yes/no response. Food assistance programs were excluded if they were uncommon (if either of the binary responses were less than 5%). Household food getting questions (Appendix C) asked participants about the frequency that they or other household members were able to obtain 26 healthy food items in the previous 30 days, which were healthier alternatives identified during the formative phase [18] and could be purchased, received from food assistance programs, received from a friend or family member, or acquired from hunting or fishing. Prepared foods from restaurants or other prepared food vendors were not included.

OPREVENT2 data collectors were either members of participating tribal communities or had the same tribal affiliation, and all data collectors were trained and certified to perform all aspects of the surveys. Surveys from three participating communities were completed in English. Data were entered using Microsoft Access, and extreme values were double-checked and reentered to minimize data entry errors. Latent class analysis (LCA) was conducted using Mplus Software version 8 (Muthén & Muthén, Los Angeles, CA, USA) [19], and bivariate analyses comparing sociodemographic variables of latent classes were conducted with Stata software version 15.1 (StataCorp, College Station, TX, USA) [20]. 

Latent class analysis (LCA) is a data-driven approach to identify underlying subgroups (“classes”) of which an overall population is comprised based on patterns of responses to a set of correlated dichotomous indicator variables [21]. The subgroups are conceptualized as not fully observable—hence “latent”—but indirectly measurable through a collection of indicators of the environment one experiences [21]. LCA uses maximum likelihood estimation to obtain the following parameter estimates: latent class probabilities (i.e., the prevalence of each subgroup/class in the sampling population) and conditional probabilities for each indicator given class membership (i.e., the probability of indicator responses within each class) [21]. Previous research has used LCA to understand the relationship between latent environmental variables and obesity, such as the food environment [22], physical activity environment [23,24], and obesogenic environments [25,26], yet this methodology has not been used to describe Native American food environments. In summary, person-centered approaches, such as LCA, provide a holistic strategy to distinguish characteristics of the food environment. 

Analysis procedure: To implement the analysis, the first step was to dichotomize the household food getting variables based on whether respondents had never or ever gotten each food item (0 and ≥1 times/month, respectively). For foods that were purchased more frequently (<10% of people reported never getting in the previous month), variables were dichotomized into the categories of weekly (0–4 times/month) and more than weekly (≥5 times/month), which was done for fresh fruit, fresh vegetables, canned vegetables, poultry, and water items. 

Next, to avoid gross overfitting, a subset of six indicators was used to conduct an exploratory LCA comparing 1-, 2-, 3-, and 4-class models. Indicators were selected to best represent aspects of the household food environments. WIC and SNAP participation items were selected based on their high utilization, and fresh fruit, fresh vegetables, game meats, and water were selected based on their importance for healthy diets. The number of classes needed to achieve within-class homogeneity was chosen based on a combination of model fit statistics, including Bayesian information criterion (BIC), Lo–Mendell–Rubin (LMR), bootstrap likelihood ratio test (BLRT) [27], the number of patterns with extreme standardized residuals (>±1.96), and model precision (based on the size of standard errors of probability estimates). For interpretation of the model fit indicators, the BLRT was given more weight, since it outperforms other model fit indicators for LCA [27]. 

After the number of classes was determined, LCA was conducted with the selected number of classes using all indicators. Participants were assigned to latent classes based on their highest posterior probability for class membership. We summarized patterns by presenting the latent class probabilities (estimated prevalence of each subgroup in the study sample) and conditional probabilities of indicator responses for each class. To further describe the latent classes using sociodemographic characteristics, we tested for similarity between classes using a significance level of α = 0.05. Since two classes were identified, the following tests were used: Pearson’s chi-squared test (categorical variables), two-sample tests of proportions (dichotomous variables), two-sample t-tests (normally-distributed, continuous variables), and Wilcoxon rank sum tests (non-normal, continuous variables). 

## 3. Results

### 3.1. Exploratory LCA Results

For the household food environment, a two-class model was found to be the appropriate number of classes for subsequent analyses (Table 1). The two-class model had a marked reduction in the number of patterns with extreme standardized residuals compared to a one-class model. While the LMR and BLRT both indicated a significantly better fit for the three-class versus a two-class model, three- and four-class models were ruled out based on a lack of substantial decrease in the number of patterns with extreme standardized residuals and reduced precision compared to a more parsimonious two-class model. Therefore, we concluded that the household food environment was comprised of two homogeneous and distinct subgroups.

### 3.2. LCA Results: “Higher” and “Lower” Access Household Food Environment Classes

**Figure 1:**
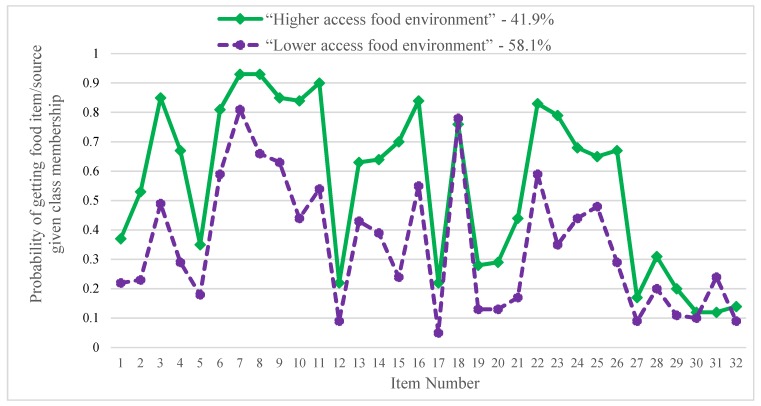
“Higher access household food environment” group has more frequent healthy food getting and food assistance participation in the previous 30 days. **Legend:** Fruits and vegetables: 1—fresh fruit *, 2—frozen fruit, 3—canned fruit (in juice), 4—canned fruit (in syrup), 5—fresh vegetables *, 6—frozen vegetables, 7—canned vegetables; grains: 8—whole wheat bread/pasta, 9—hot cereal (like oatmeal), 10—low-sugar, high-fiber cereals (like shredded wheat), 11—high-fiber rice (like wild or brown); proteins: 12—poultry *, 13—game meat, 14—seafood/fish, 15—low-fat deli meat, 16—beans/peas; drinks: 17—skim milk, 18—low-fat milks (1 or 2%), 19—milk alternatives (like almond), 20—water *, 21—sugar-free drinks (like club soda), 22—100% juice; other: 23—low-fat/sugar snacks (like baked chips), 24—dried fruit/nuts, 25—cooking spray, 26—light or low-fat dressings; food assistance programs: 27—Special Supplemental Nutrition Program for Women, Infants, and Children (WIC), 28—Supplemental Nutrition Assistance Program (SNAP), 29—commodity foods (Food Distribution Program for Indian Reservations (FDPIR)), 30—food bank, 31—senior center meals, 32—church. * Commonly gotten foods were dichotomized as getting more than weekly (5 or more times monthly) or up to weekly (0–4 times per month).

The household food environment can be described by two subgroups with different patterns of access to and use of the household food environment: the “higher access household food environment” (class 1) and “lower access household food environment” (class 2). Conditional probabilities of food getting and food assistance participation are shown in Figure 1 and Table 2. Class 1 was named the “higher access household food environment” group, as its members tended to have higher conditional probabilities for getting each food item and for participating in food assistance programs compared to the “lower access household food environment” group, except for low-fat milks and participation in food bank and senior center meal programs. The majority (58.1%) of participants were classified as belonging to the “lower access household food environment” group. 

Participants in the “higher access” group reported more frequently getting fruit and vegetables (items 1–7). Fresh fruit and fresh vegetables were low across both groups (22–37% and 18–35% for fresh fruit and fresh vegetables, respectively). Canned fruit items (stored in juice and syrup) were frequently gotten among the “higher access household food environment” group; 67% and 85% of the “higher access” group reported getting canned fruit in juice or canned fruit in syrup, respectively. Similarly, the two access subgroups had different access to whole grains (items 8–11); the “higher access” group reported high probability (84–93%) of getting grain items like whole wheat bread or pasta, hot cereals (like oatmeal), low sugar high fiber cereals, and high fiber rice (like wild or brown rice) compared to moderate probability (44–66%) of getting these grain items among the “lower access” group. High fiber rice (including wild and brown rice) was frequently gotten among the “higher access” group (90%) compared to the “lower access” group (54%). 

The “higher access” group also reported frequently getting (22–84%) of various proteins (items 12–16) compared to the “lower access” group (5–55%). Access to game meats was higher in the “higher access” group, with 63% of participants in the “higher access” group reporting ever getting game meats in the previous 30 days compared to 43% in the “lower access” group. The “higher access” group also reported more access to drinks (items 17–22), including milks (skim and alternative), water, sugar-free drinks, and 100% fruit juice; however, getting low-fat milks was similar across both groups. The “higher access” group reported more access to other items (items 23–26), including low fat or low sugar snacks, dried fruit or nuts, cooking spray, and light or low-fat dressings. Lastly, food assistance participation was similar across the “lower” and “higher” access groups (items 27–32).

### 3.3. Results of Bivariate Analyses

Table 3 describes the demographic characteristics of each subgroup of the household food environment. The “lower access household food environment” group had significantly higher mean age as well as smaller mean household size and fewer children per household than the “higher access” group. Participants in the “lower access” group were more likely to have less than a high school diploma and have a bachelor’s or graduate degrees; however, these results fell short of the traditional significance threshold (*p* = 0.061). There were no significant differences based on gender, employment status, marital status, or BMI between the household food environment classes. 

### 3.4. Results of Sensitivity Analyses

Several sensitivity analyses were conducted to establish the stability of the identified classes. First, we verified that the frequencies of patterns with extreme standardized residuals (>±1.96) were rare (represented <1% of the data sample). Second, we independently tested the associations of indicators on which classes were not well distinguished with BMI. Third, we assessed BMI variation in a three-class model and found that this was not significant. Fourth, we assessed the generalizability of findings across the three study communities. Comparable class distributions were found in the individual communities as in the overall sample.

## 4. Discussion

This analysis is the first to use a person-centered approach (LCA) to describe the household food environment of three Native American on-reservation communities in the Midwest and Southwest. This methodology allowed for a holistic analysis of the household food environment, including market foods, traditional foods, and food assistance programs, which are important aspects of Native American food environments. Since our analysis examined food getting regardless of food source, this analysis reflects food environments both on- and off-reservation. Community partners and experts have reported that community members commonly travel long distances to purchase food. This is common for Native Americans living on-reservation and agrees with previous literature describing the lack of healthy foods and grocery stores on reservations, necessitating long drives to access grocery stores [10,11,13,14,28]. A strength of this work is that we are not limiting food getting to on-reservation sources, which would miss the large portion of off-reservation food getting. From this analysis, we identified two subgroups related to food access environments and described the demographic characteristics of these subgroups. To discuss the significance of our findings, we will highlight five key findings of this study and compare to the broader literature.

From this analysis, we concluded that the household food environments of rural Native American communities can be described using subgroups reflecting higher and lower food access, as demonstrated by the results of our exploratory LCA. This means that LCA was a useful methodology for describing complex Native American household food environments. Because we conceptualized the household food environment using food getting (which incorporated traditional food items and ways of food getting) and food assistance indicators, our analysis reflected both the local market and traditional food environments as well as the food environments of nearby communities. This enriches the previous research describing Native American food environments, which found that fewer healthy food outlets were present on or near tribal communities compared to non-tribal communities [11] and that healthy food items were less frequently available [12,13] and more expensive on-reservation compared to off-reservation [12]. Since Native Americans living on reservations typically travel more than 30 miles to get affordable, healthy foods [28], this analysis may more accurately reflect the food environments that people experience. This research also adds to the literature describing methods for assessing the food environment. The Perceived Nutrition Environment Measures Survey (NEMS-P) also aims to holistically describe the perceived food environment by examining three dimensions: community, consumer, and home [29]. While having a methodology that can assess the food environments is useful to understand the importance of exposures across populations, there are some important limitations of using these instruments to assess Native American food environments. First, the NEMS-P was developed in an urban population of Philadelphia, which did not include Native American populations and so the food environments of rural, Indigenous populations are not reflected in the development of the survey [29]. Second, due to the unique history of Indigenous populations, an analysis of the Native American on-reservation food environments must include food assistance and traditional foods to promote wellness and equity, neither of which are captured by NEMS-P. Third, the use of LCA allowed for the correlation between items and we believe it was a particularly useful methodology for understanding the latent food environment through analyzing the patterns of responses to highly correlated indicator variables [21]. Because of these limitations, we think that this paper complements the efforts by Glanz et al. to describe food environments across populations by looking at population-specific approaches [30]. The results of this analysis suggest that LCA is a useful methodology to understand this complexity for Native American populations living on reservations. Future research should examine the utility and adaption of NEMS-P for assessing Native American food environments to facilitate cross-population comparisons. 

Second, we found low fruit and vegetable access among both the higher and lower access household food environments, while indicators for traditional food access varied between these groups. For example, wild or brown rice and game meats were frequently gotten among higher access groups. Increasing access to traditional foods may be an important strategy to improve food security for lower access groups. Previous studies have demonstrated the importance of traditional foods in promoting the health and wellness of Indigenous people [5,31] and Native American Nations are increasingly interested in promoting food sovereignty [32]. Reeds et al. documented three dietary patterns based on traditional food and processed food consumption and found that type 2 diabetes incidence was associated with the pattern with the highest processed food consumption [33]. Regarding fruit and vegetable access, previous research has found that convenience and dollar stores located on or near reservations had less fruit and vegetables available compared to grocery stores [13] and that most Native Americans did not have adequate daily intake [34]. Addressing the reduced access and intake of fruits and vegetables is important to understanding opportunities for preventing diet-related chronic diseases. Native American Nations are advocating for their food sovereignty and the 2018 Farm Bill has included more efforts to promote locally grown produce and traditional foods [35].

Third, the least distinguishing factor between the lower and higher access household food environment subgroups was food assistance program participation, indicating that these programs are not adequately addressing the lack of food access on reservations and that additional policy, systems, and environmental changes are needed to promote food access. While these programs are vital to improving the nutritional status of low-income Americans, they are increasingly aiming to address obesity [36] and to meet dietary guidelines [37] of the populations they serve. Previous research on food assistance programs has yielded mixed results on whether they have increased access and intake of healthier foods for Native Americans. Similar to our findings, Bauer et al. described an association between household food insecurity and SNAP participation [38]. Previous research found that WIC-participating stores on reservations stocked healthier foods compared to non-WIC stores [12]. Shanks et al. also found that FDPIR packages were not meeting 2010 federal dietary guidelines based on healthy eating index (HEI-2010) scores [16]. This paper is the first known analysis to incorporate food assistance programs along with other food access indicators to understand the household food environment of Native American communities; future research should similarly include food assistance in analyses of Native American food environments.

Lastly, the lower access group had significantly higher mean age as well as smaller household size and fewer children per household than the higher access group; however, BMI did not significantly vary between these subgroups. A potential explanation for these demographic differences between higher and lower access household food environments is the important role of poverty in limiting healthy food getting. A recent meta-analysis of six cohort studies of older adults found an association between increasing age and elevated risk of malnutrition [39]. This meta-analysis also found a higher risk of malnutrition among single, non-widowed participants compared to married participants [39]. Previous research has documented higher food insecurity prevalence among single-person and single-parent households in Ontario, Canada, largely due to higher poverty of these groups [40]. Another study examining the USDA definition of food insecurity saw that higher food insecurity was found in single-person households and female-headed households compared to married couple households [41]. Based on WIC program eligibility, an explanation for the lower access group also having fewer children per household could be that food access decreases once food program benefits that go along with having children are no longer there. This analysis also adds to the growing research on Native American food environments and relationship to adiposity. More recently, Love et al. found that frequent shopping at convenience stores, gas stations, or dollar stores as regular food sources was associated with an increased prevalence of obesity and diabetes [15]. Previous studies describing Native American food environments [12,13] did not examine the relationship between the food environment and BMI, although Jernigan et al. described an association between food insecurity of Native American participants and higher prevalence of obesity, diabetes, and hypertension [42]. To our knowledge, no studies have examined the association between Native American food environments and food insecurity, and this could be explored in future research.

This study has several limitations to consider. First, we used cross-sectional data to understand the association between household food environment subgroups and BMI, and so, future longitudinal studies must be conducted to establish temporality. Second, this study examined the relationship between distal variables: household food environments and BMI, which may have contributed to nonsignificant findings. Future work could examine the relationship between latent classes of household food environments and more proximal behavioral outcomes such as dietary habits, quality, and intake. Third, our indicators for the household food environment focused on food getting and food assistance participation and did not account for the quality and price of healthy foods. Instruments like Nutrition Environment Measures Survey in stores (NEMS-S) have been adapted to capture such characteristics of food environments for use in Native American settings [12]. Fourth, we did not directly measure food outlets on or near reservations. Future studies could additionally include geographical-based assessments of food outlets, similar to Fleischhacker et al. [43], to complement results using this methodology.

## 5. Conclusions

From this analysis, we conclude that LCA was a useful methodology to describe the complexity of Native American household food environments by including indicators from market foods, traditional foods, and food assistance programs. When comparing indicators between these classes, we found low fruit and vegetable access for both “higher” and “lower access household food environments” and little difference in food assistance program participation, while indicators for traditional food access varied between these groups. After examining the demographic characteristics between classes, we found that the “lower access” group had significantly higher mean age, smaller household size, and fewer children per household than the “higher food access” group; however, BMI did not significantly vary between these subgroups.

This research is the first to explore and describe the household food environments of three Native American communities from the Midwest and Southwest Regions of the US using a person-centered approach and a diverse set of household food environment indicators. We found evidence of two distinct, latent subgroups (“lower access” and “higher access”) of the household food environment that were consistent across three Native American communities, highlighting the need for further research to better characterize the unique aspects of Native American household food environments to identify potential levers for health promotion with Native American Nations and communities.

## Figures and Tables

**Table 1:** Model fit indicators for 1-, 2-, 3-, and 4-class models using data from three Native American communities (*n* = 300) from the Midwest and Southwest, 2016–2017 *.

Latent Variable	Number of Classes	#S	BIC	LMR	BLRT	#SR
Household food environment	1	6	1996.491	N/A	N/A	15
	2	13	1904.045 ^a^	0.0000 ^b^	0.0000 ^b^	5
	3	20	1920.240	0.0358 ^b^	0.0000 ^b^	4
	4	27	1947.860	0.0615	0.6000	2

* Model fit statistics based on exploratory latent class analysis using a subset of indicators for each latent variable; #S—number of free parameters, BIC—Bayesian information criterion, LMR—Lo–Mendell–Rubin, BLRT—bootstrap likelihood ratio test, #SR—number of patterns with standardized residuals >±1.96; ^a^ the lowest BIC value for the latent variable, ^b^ in LMR/BLRT, fits significantly better than a k-1 class model.

**Table 2:** Probability of healthy food getting, and food assistance participation given household food environment class membership among three Native American communities (*n* = 300) from the Midwest and Southwest, 2016–2017.

Indicator Probability (Standard Error)		Class 1 “Higher Access Household Food Environment”	Class 2 “Lower Access Household Food Environment”
Category	Class Size	41.9%	58.1%
Fruit and Vegetables	1. Fresh fruit ^1,2^	0.37 (0.07)	0.22 (0.05)
	2. Frozen fruit	0.53 (0.06)	0.23 (0.05)
	3. Canned fruit in 100% juice	0.85 (0.05)	0.49 (0.05)
	4. Canned fruit in light/heavy syrup	0.67 (0.07)	0.29 (0.05)
	5. Fresh vegetables ^1,2^	0.35 (0.07)	0.18 (0.05)
	6. Frozen vegetables	0.81 (0.06)	0.59 (0.05)
	7. Canned vegetables	0.93 (0.04)	0.81 (0.04)
Grains	8. Whole wheat bread or pasta	0.93 (0.04)	0.66 (0.04)
	9. Hot cereal, like oatmeal	0.85 (0.05)	0.63 (0.05)
	10. Low-sugar, high-fiber cereals	0.84 (0.06)	0.44 (0.05)
	11. High-fiber rice, like wild or brown	0.90 (0.05)	0.54 (0.05)
Proteins	12. Poultry ^1^	0.22 (0.05)	0.09 (0.04)
	13. Game meat ^2^	0.63 (0.07)	0.43 (0.05)
	14. Seafood or fish	0.64 (0.06)	0.39 (0.04)
	15. Low-fat deli meat	0.70 (0.08)	0.24 (0.05)
	16. Beans or peas	0.84 (0.04)	0.55 (0.05)
Drinks	17. Skim milk	0.22 (0.05)	0.05 (0.02)
	18. Low-fat milks, like 1 or 2%	0.76 (0.05)	0.78 (0.04)
	19. Milk alternatives, like almond	0.28 (0.05)	0.13 (0.04)
	20. Water ^1,2^	0.29 (0.06)	0.13 (0.04)
	21. Sugar-free drinks, like club soda	0.44 (0.07)	0.17 (0.04)
	22. 100% fruit juice	0.83 (0.05)	0.59 (0.05)
Other	23. Low-fat or low-sugar snacks, like baked chips	0.79 (0.07)	0.35 (0.06)
	24. Dried fruits or nuts	0.68 (0.06)	0.44 (0.05)
	25. Cooking spray	0.65 (0.05)	0.48 (0.05)
	26. Low-fat or light dressings	0.67 (0.06)	0.29 (0.05)
Food Assistance	27. WIC ^2^	0.17 (0.04)	0.09 (0.03)
	28. SNAP ^2^	0.31 (0.05)	0.20 (0.03)
	29. Commodity Foods or FDPIR	0.20 (0.04)	0.11 (0.03)
	30. Food Bank	0.12 (0.03)	0.10 (0.03)
	31. Senior center meals	0.12 (0.04)	0.24 (0.04)
	32. Church	0.14 (0.04)	0.09 (0.02)

^1^ Commonly gotten foods were dichotomized as getting more than weekly (5 or more times monthly) or up to weekly (0–4 times per month). ^2^ Indicators used for exploratory latent class analysis (LCA).

**Table 3:** Comparing demographic characteristics of two household food environment classes.

Demographic Mean or % Estimate (SD/*n*) ^1^		Overall	Household Food Environment	
			Class 1	Class 2
Class name		N/A	“Higher food access”	“Lower food access”
Sample size (%)		300	125 (41.7)	175 (58.3)
Mean age in years (SD)		46.8 (13.9)	44.6 (13.1) *	48.3 (14.2) *
% Female (*n*)		74.0 (222)	73.6 (92)	74.3 (130)
Mean household size (SD)		3.4 (1.8)	3.7 (1.8) *	3.2 (1.7) *
Mean number of children in the household (SD)		1.2 (1.4)	1.6 (1.6) *	1.0 (1.2) *
Employment status				
	% Unemployed, retired, or disabled (*n*)	22.0 (66)	18.4 (23)	24.6 (43)
	% Student (*n*)	3.3 (10)	5.6 (7)	1.7 (3)
	% Part time employee, seasonal or temporary (*n*)	12.3 (37)	14.4 (18)	10.9 (19)
	% Full time employment (*n*)	62.3 (187)	61.6 (77)	62.9 (110)
Highest attained education				
	% Less than high school (*n*)	11.0 (33)	8.0 (10)	13.1 (23)
	% High school diploma/General education development degree (*n*)	33.7 (101)	32.8 (41)	34.3 (60)
	% Some post-secondary (*n*)	48.0 (144)	55.2 (69)	42.9 (75)
	% Completed bachelor’s or graduate degree (*n*)	7.3 (22)	4.0 (5)	9.7 (17)
Marital status				
	% Single/separated/widowed/divorced (*n*)	67.9 (203)	65.3 (81)	69.7 (122)
	% Married/common law/lives with partner (*n*)	32.1 (96)	34.7 (43)	30.3 (53)
Mean BMI in kg/m^2^ (SD)		31.2 (6.2)	31.6 (6.6)	31.0 (5.9)

^1^ %—percent; SD/*n*—standard deviation of the mean or numerator of percentage; SD—standard deviation; BMI—body mass index. * statistically significant at the α = 0.05 level.

## Appendix A. Data Availability

Due to the data use agreements with participating communities and respect for tribal sovereignty, we are unable to make data directly available to readers. All data requests would need to be approved by relevant tribal authorities prior to distribution of deidentified data.

## Appendix B. Food Assistance Section

Question: Do you or a household member receive any of the following types of food assistance?
1. WIC (Women, Infants, and Children) ____ Yes_1_ ____ No_0_
2. SNAP (Supplemental Nutrition Assistance Program, formerly known as food stamps) ____ Yes_1_ ____ No_0_
3. Commodity foods, like FDPIR (Food Distribution Program on Indian Reservations) ____ Yes_1_ ____ No_0_
4. Food Bank ____ Yes_1_ ____ No_0_
5. Senior center meals ____ Yes_1_ ____ No_0_
6. Church ____ Yes_1_ ____ No_0_
7. Other tribal food distribution programs ____ Yes_1_ ____ No_0_
8. Local farm surplus ____ Yes_1_ ____ No_0_
9. Summer Food Service Program (lunch meals for children) ____ Yes_1_ ____ No_0_
10. Other, please specify: ______________________________________________________________

## Appendix C. Household Food Getting Frequency Section

Question: In the last 30 days, how often did you get the following?	Number of times:
1. Fresh fruits	____
2. Frozen fruits	____
3. Canned fruits or fruit cups in 100% juice	____
4. Canned fruits or fruit cups in light or heavy syrup	____
5. Fresh vegetables or greens	____
6. Frozen vegetables, not including potatoes like French fries or tater tots	____
7. Canned vegetables	____
8. Beans and peas, canned or dried, not including green beans	____
9. Dried fruits and nuts, including trail mix	____
10. 100% whole wheat breads or pastas	____
11. Hot cereals, like Cream of Wheat, oatmeal, or atole	____
12. Low-sugar, high-fiber cereals, like shredded wheat	____
13. High-fiber rice, like brown rice or wild rice	____
14. Poultry, like chicken or turkey	____
15. Game meat, like venison, elk, buffalo, or moose	____
16. Fish or seafood	____
17. Low-fat or low-sugar snacks, like baked chips, graham crackers, or low-sodium pretzels	____
18. Low-fat deli meats, like low-fat bologna or turkey	____
19. Cooking spray, like Pam	____
20. Low-fat milks, like 1% or 2%, including Lactaid	____
21. Skim (fat-free) milk, including Lactaid	____
22. Other milk, like soy, rice, almond, or coconut	____
23. Sugar-free drinks, like sparkling water	____
24. Water, including plain, tap, or bottled	____
25. 100% fruit juice	____
26. Low-fat or light dressings, like Miracle Whip and light mayo	____
